# Seroprevalence and risk factors of brucellosis in pastoralists and their livestock in Central Equatoria State, South Sudan

**DOI:** 10.1371/journal.pntd.0012144

**Published:** 2024-12-19

**Authors:** Emmanuel P. Lita, Erneo B. Ochi, Gerald Misinzo, Henriette van Heerden, Robab Katani, Jacques Godfroid, Coletha Mathew

**Affiliations:** 1 Department of Veterinary Medicine and Public Health, College of Veterinary Medicine and Biomedical Science, Sokoine University of Agriculture, Morogoro, Tanzania; 2 School of Veterinary Medicine, University of Juba, Juba, South Sudan; 3 SACIDS Africa Centre of Excellence for Infectious Diseases, SACIDS Foundation for One Health, Sokoine University of Agriculture, Morogoro, Tanzania; 4 Department of Microbiology, Parasitology and Biotechnology, College of Veterinary Medicine and Biomedical Sciences, Sokoine University of Agriculture, Morogoro, Tanzania; 5 Department of Veterinary Tropical Diseases, University of Pretoria, Faculty of Veterinary Science, Onderstepoort, South Africa; 6 The Huck Institute of the Life Sciences, Pennsylvania State University, University Park, Pennsylvania, United States of America; 7 The Nelson Mandela African Institution of Science and Technology, Arusha, Tanzania; 8 Department of Arctic and Marine Biology, Faculty of Biosciences, Fisheries and Economics, UiT-The Arctic University of Norway, Tromsø, Norway; 9 Department of Veterinary Anatomy and Pathology, College of Veterinary Medicine and Biomedical Sciences, Sokoine University of Agriculture, Morogoro, Tanzania; Institute of Continuing Medical Education of Ioannina, GREECE

## Abstract

**Background:**

Brucellosis poses serious public health implications and substantial economic losses in pastoral rural settings in South Sudan. In humans, brucellosis is almost always originating from animals. Current literature provides scant data regarding the seroprevalence of brucellosis in South Sudan. This cross-sectional study investigates the seroprevalence of brucellosis among the pastoral community and livestock and identifies risk factors for the disease from two Counties, Terekeka and Juba in Central Equatoria State (CES), South Sudan.

**Methodology:**

A total of 986 sera; from humans (n = 143), cattle (n = 478), sheep (n = 86), and goats (n = 279) were randomly collected from 17 cattle camps in CES. Sera for the humans, cattle and goats were screened for *Brucella-*specific antibodies using Rose Bengal plate test (RBPT) and further confirmed by competitive enzyme-linked immunosorbent assay (c-ELISA) in series due to the cost of testing. All the sera from sheep were tested in parallel using RBPT and c-ELISA as the sheep samples were few and were all tested negative on the RBPT. A camp was considered positive when at least one animal of either species tested positive on the c-ELISA. A structured questionnaire was used to collect information on potential individual and herd level risk factors. Univariate analysis using binary logistic regression with a confidence interval of 95% at a p-value of ≤ 0.05 was used to identify the association between the potential individual risk factors and *Brucella* seropositivity. The investigated risk factors for livestock included age, sex, species, prior abortion history, retained placenta, parity, and reproductive status. Variables found to have associations in univariate analysis (*p* = 0.25) with *Brucella* seropositivity were further included in multivariable logistic regression. The risk factors investigated for humans included, gender, age, educational level, occupation, marital status, drinking of raw milk, aiding female animals during delivery, eating undercooked meat and blowing of air into the cow’s uterus through the vagina, a practice in South Sudan.

**Results:**

The study revealed seroprevalence of 21.7%, 11.8%, and 4.8% in cattle, goats, and humans, respectively. Our results indicated that all sheep serum samples were negative on both RBPT and c-ELISA. The seropositive in the 13 camps from Terekeka County was 100.0% (13/13) compared to 50.0% (2/4) seropositive from 4 camps in Juba County. All the variables investigated in the univariate analysis of risk factors in cattle were significantly associated with *Brucella* seropositivity: sex (OR:4.5, 95% CI: 2.2–8.9, *p*<0.001), age (OR:6.6, 95% CI: 2.3–19.1, *p*:<0.001), abortion history (OR:3.1, 95% CI: 1.8–5.2, *p*:<0.001), retained placenta (OR:2.5, 95% CI: 1.4–4.4, *p* = 0.001), parity (OR:2.3, 95% CI: 1.1–4.7, *p* = 0.020), However, in small ruminants, none of the potential risk factors were associated with *Brucella* seropositivity. In humans, blowing air through a cow’s vagina (OR: 1.4, 95%CI: 0.782–2.434, *p* = 0.035) was the only variable found to be significantly associated with *Brucella* seropositivity in the univariate analysis. The forceful blowing of air into a cow’s vagina to induce milk letdown is a common practice among the pastoral communities in South Sudan.

The multivariable logistic regression model identified sex, age, and abortion history as statistically significant factors for *Brucella* seropositivity in cattle. The odds of seropositivity were nearly threefold (OR: 2.8; 95% CI: 1.3–5.8, *p* = 0.006) higher in cows compared to bulls (male cattle). Cattle over two years old had higher odds of *Brucella* seropositivity than young animals (OR: 3.5, 95% CI: 1.2–10.3-, *p*: 0.025). Cows with a history of abortion had higher odds of *Brucella* seropositivity (OR: 2.8, 95% CI: 1.6–4.7, *p* = 0.001).

**Conclusion:**

This study reports the occurrence of brucellosis in goats and its absence in sheep in (CES), South Sudan. The present study also shows the occurrence of brucellosis in cattle, goats and people in the pastoral community and recommends for the implementation of the One Health approach and awareness campaigns for effective mitigation of this disease.

## Author summary

Brucellosis is a neglected, bacterial zoonotic disease that is caused by several species of the genus *Brucella*. Cross-species transmission of *Brucella* can occur in mixed or integrated farming systems. The disease poses serious public health implications and substantial economic losses particularly in low-income countries including South Sudan.

This study was conducted to estimate the seroprevalence of brucellosis in pastoralists, and their livestock as well as identify potential risk factors associated with *Brucella* infection. Knowledge of the seroprevalence of brucellosis and risk factors is a prerequisite towards planning an effective mitigation strategy for the disease.

The study revealed high seroprevalence of brucellosis in cattle compared to goats, and the following risk factors were identified; prior history of abortion, age (old) and sex (female) significantly associated with *Brucella* infection. Surprisingly, sheep were found to be seronegative.

The results of this study showed that brucellosis is prevalent in cattle, goats and people in the pastoral community in CES. Hence, the study suggests the need for implementing control measures such as vaccination and regular screening of cattle and goats to reduce production loses, raising public awareness and knowledge about brucellosis.

## Introduction

Brucellosis is a significant zoonotic disease affecting many countries in sub-Saharan Africa including South Sudan. *Brucella* spp. are the aetiological agents of the disease that affect both humans and animals [1]. The species of *Brucella* are well-adapted to their hosts, however, accidental transmission due to management conditions to secondary hosts such as humans have been reported [2].The disease affecting livestock and humans is caused by *B*. *melitensis* mainly in goats and sheep, *B*. *abortus* mainly in cattle and buffaloes, and *B*. *suis* in pigs [3].There are several predisposing factors attributing to the occurrence of brucellosis in humans and animals.

In livestock, the disease causes reduced milk production, longer calving intervals, abortions, stillbirth, swollen joints and infertility [4]. Transmission of brucellosis to humans occurs through the consumption of infected, unpasteurized animal milk products, through direct contact with infected animal parts (such as the placenta by infection through bruised skin and mucous membranes), and inhalation of infected aerosolized particles [5]. In humans, clinical brucellosis presents as acute or sub-acute febrile illness and is characterized by intermittent fever accompanied by malaise, anorexia, and prostration [6].

The economic losses due to brucellosis are enormous and incur costs to humans either directly (e.g. health care costs for the diagnosis, treatment, and management of clinically ill patients) or indirectly (e.g. loss of work days, lost leisure time, loss of productive years due to premature death [7]. Studies have reported varying seroprevalences of brucellosis ranging from 1.2% - 6.8% in sheep, 0.3% - 23.1% in goats, 1.2% - 30.8% in cattle and 4.4% - 10.8% in humans in the pastoral and mixed farming systems where humans have been embedded with livestock, so it constitutes a high risk of infection [8–13]. Furthermore, several studies within the African region have identified several risk factors which include but not limited to; management systems, age, sex, species, environment, herd size, agroecology, and reported varying prevalence levels of brucellosis based on spatial and temporal features, diagnostic methods, and species [8,9,14–16].

However, few studies have been conducted in South Sudan to assess the prevalence of brucellosis in humans and cattle [11,17–20]. These studies have reported seroprevalence ranging from (23.2% - 31.1%) and (32.1% - 44.0%) in cattle and humans, respectively. There are no reported studies on brucellosis in sheep and goats. In South Sudan, the pastoral communities usually keep their livestock such as cattle, sheep, and goats under extensive in the cattle camps [21,22]. In CES, a large percentage of goats are kept as part of mixed herds with cattle, and therefore the same factors that have affected cattle populations also may effect on goats population [21]. It is found that keeping different animal species plays a pivotal role in cross-species transmission and maintenance of brucellosis [23–25]. There is inadequate knowledge of the epidemiology and risk factors of brucellosis in small ruminants and their role in the transmission of the infection to humans in South Sudan. Understanding these gaps in knowledge is a prerequisite for the development of effective mitigation measures for the disease in South Sudan.

Hence, this study estimates seroprevalence and identifies risk factors associated with *Brucella* seropositivity among pastoralists and their cattle, sheep, and goats in Central Equatoria State (CES), South Sudan.

## Materials and methods

### Ethics statement

The study protocol was approved by the Institutional Review Board of Sokoine University of Agriculture under reference number (DPRTC/R/186/16) and the National Ministry of Health Research Ethics Review Board (RERB-P No: 13/14/02/2023), South Sudan Additionally, permissions for data collection were obtained from the State Ministry of Animal Resources, Fisheries and Tourism and the Ministry of Health, CES, South Sudan. Moreover, Export and Import permits for shipment of the biological samples were obtained from the National Ministry of Livestock and Fisheries, South Sudan, and the Ministry of Livestock and Fisheries, United Republic of Tanzania. An oral consent was obtained from each study participant who agreed to participate in the study prior to data collection.

### Study area

The study was purposively conducted in Terekeka and Juba counties of CES, South Sudan. These counties has large livestock population in the region [21]. Terekeka is one of the six counties in CES with an area size of 10,538.23 km^2^ [26]. It lies on both the east and west banks of the White Nile River. The County includes low-lying swampy areas that usually flood but provide grazing in the dry season. Rainfall is about 900 millimetres annually. The main inhabitants of Terekeka are the Mundari people. Livestock rearing is considered an important part of people’s livelihood in Terekeka County, CES. There are ten payams in Terekeka County; Terekeka, Gameiza, Nyori, Mangala North, Muni, Reggo, Rijong, Tali, Tombek and Tindilo. Three payams namely: Reggo, Nyori and Terekeka were selected due to the presence of huge cattle camps as indicated by the County veterinary officer. Furthermore, a list comprised 23 known cattle camps in Terekeka, Reggo and Nyori payams was provided by the County’s Animal Health Department. Thirteen camps were then randomly selected from the list. A proportional random sampling was then used to sample individuals.

**Fig 1:**
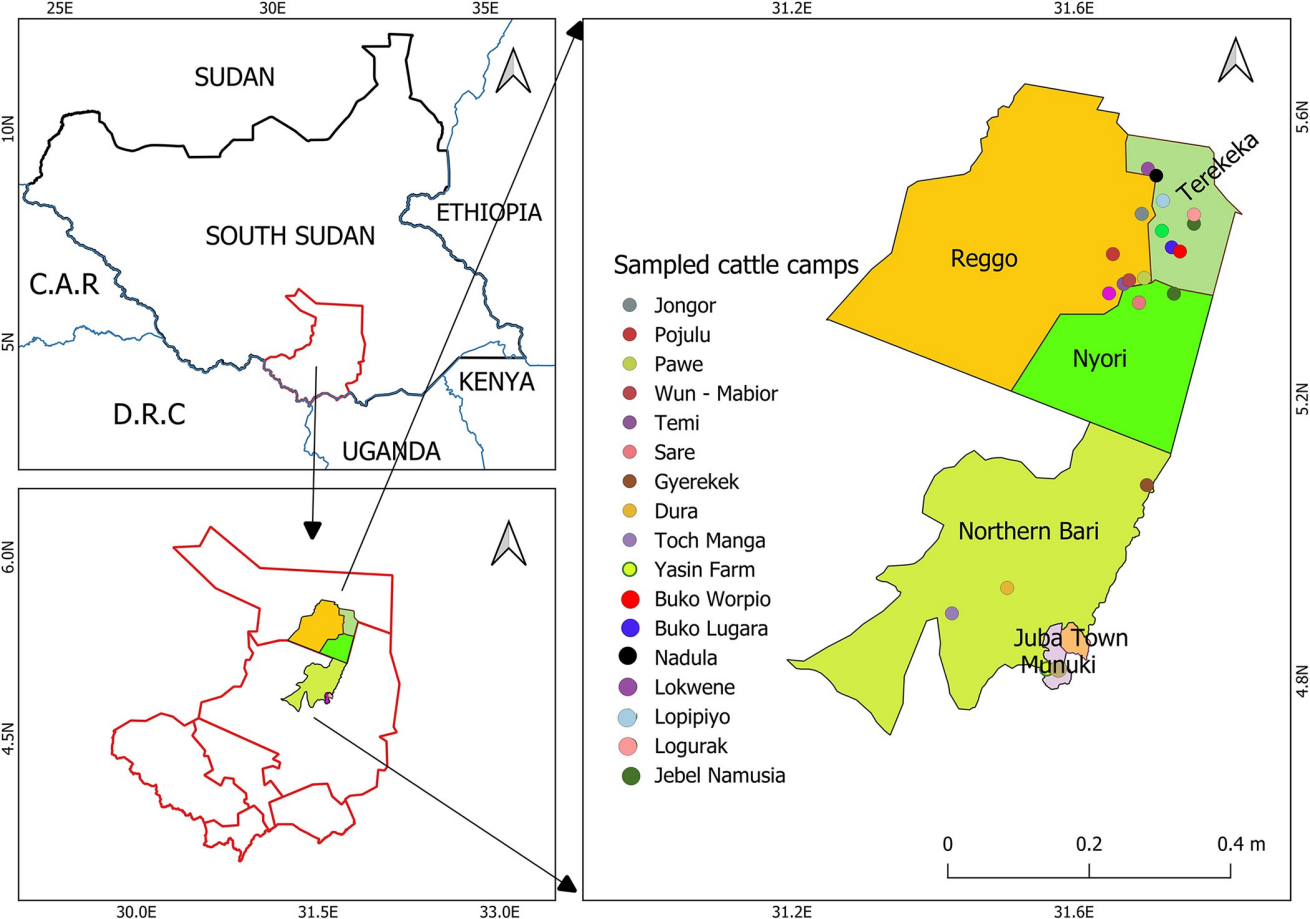
Location of the Central Equatoria State of South Sudan indicated in upper left with study area showing sampled cattle camps in Terekeka and Juba Counties in lower left consisting of Reggo, Terekeka and Nyori in the Terekeka County and Northern Bari and Munuki Payams in the Juba County, indicated on right. Map generated using QGIS software version 3.32.1. Shapefiles for administrative boundaries from Humanitarian Data Exchange https://data.humdata.org/dataset/cod-ab-ssd.

Juba County is located in the centre of CES and hosts the capital city of South Sudan. The County covers an area size of 18,396.15 km^2^ [26]. It borders Terekeka County to the north and Kajo-keji and Lainya counties to the South. Unlike Terekeka County, residents of Juba engage in a diverse range of livelihood. Juba County has 13 counties, out of which two namely; northern Bari and Munuki payams were purposively selected due to availability of cattle camps. There are few cattle camps in Juba County due to the cattle raiding and insecurity [21]. A list comprised nine known cattle camps in Juba was provided by the County’s Animal Health Department. Out of that, four cattle camps were randomly selected from the two payams in Juba County as shown in Fig 1. A proportionate random sampling was then used to select the individual animals from each selected camp.

### Study design and subject

A cross-sectional design was planned using a multistage method of sampling for data collection. Briefly, a list comprised names of known cattle camps in the selected payams from each county was provided by the County’s Animal Health Departments. The camps were then randomly selected and a proportional random sampling was then used to sample the individual animals. Generally, cattle camps comprised several herds which are owned by different families ranging from 30–80 depending on the location and the size of the camp [21]. The number of animals in a herd ranges from 15–50 heads in small herd and 51–200 heads for a medium herd The study subjects comprised humans linked to cattle, ≥18 years old and of different genders. Livestock species, including cattle, sheep, and goats were sampled from ≥ 6 months old, and from different sexes. These animal species had no previous record of brucellosis vaccination and were mixed in the same cattle camps and managed entirely under a pastoral farming system.

### Sample size determination in animals and humans

The total sample required was determined according to the formula given by [27]. An expected individual animal prevalence (P) of 25.3% [20] and (P) of 50.0% was used for calculating the sample size of cattle and small ruminants, respectively. The 50.0% expected prevalence for small ruminants was chosen because no previous studies were conducted to estimate the prevalence of brucellosis in goats and sheep in the context of South Sudan.

The sample size for the small ruminants (sheep and goats) was calculated collectively due to few numbers of sheep compared to goats in the cattle camps [21]. Although different species of livestock are kept together in the cattle camps in CES, only that there are few sheep kept compared to other livestock. This is due to the taboos or cultural practices related to both keeping of sheep and consumption of its products among the indigenous communities [21]. Based on the formula, 384 samples were to be collected from small ruminants and 290 from the cattle. However, a total of 478 cattle, 86 sheep and 279 goats of different ages and sexes were included in this study. In humans, a total of 143 blood samples were collected from herders in the selected cattle camps who participated voluntarily. As indicated by [22] a herd of 100 cattle requires up to 3 herders to manage. Therefore, it is obvious to sample few humans from the cattle camps as it is the case in this study.

### Blood collection and seroprevalence

A total of 5 mL of blood from small ruminants and 7 mL from cattle were drawn aseptically from the jugular vein of each randomly selected animal using a needle and plain vacutainer tube. Immediately, the vacutainer tubes were labelled, coded and kept at room temperature overnight. The next day, the samples were centrifuged for 5 minutes at 3,000 rpm, and sera were harvested and placed into labeled cryovial tubes of 2 mL. A case history, detailed information about each animal sampled, and metadata were recorded in the datasheet. In humans, a registered medical technician drawn 5mL of blood from the cephalic vein, and sera separation followed the same protocol used in the animals. The collected sera were kept at -20°C pending analysis.

### Rose Bengal plate test (RBPT)

The harvested sera from the humans, cattle, sheep and goats were all screened for *Brucella* antibodies using RBPT according to the procedure described by [3].The sera from the humans, cattle and goats were tested in series using RBPT and c-ELISA due to the cost. In contrast, sheep sera were tested in parallel using RBPT and c-ELISA. The parallel testing of the sheep sera was aimed at improving the negative predictive value (NPV) as all the sera were tested negative on RBPT. The antigen was obtained from the Animal and Plant Health Agency (APHA), New Haw, Addlestone, Surrey, England.

A cattle camp was considered positive if at least one positive *Brucella* reactor was found among the animals.

The test was conducted at the College of Veterinary Medicine and Biomedical Science, Sokoine University of Agriculture, Morogoro, Tanzania.

### Competitive enzyme linked immunosorbent assay (c-ELISA)

Sera for cattle, goats and humans found positive on the RBPT were further subjected to a c-ELISA kit (Boehringer Ingelheim Svanova, Uppsala, Sweden) for confirmation. The test was performed according to the protocol provided by the manufacturer with positive and negative controls. The samples were run in duplicates.

### Questionnaire administration

A structured questionnaire was prepared and administered to 143 respondents who were in close contact with the animals. The respondents participated voluntarily in the study. Data on age, sex, parity, abortion history, retained placenta, reproductive status were collected. Moreover, data on practices such as consumption of raw milk and meat, blowing through vagina and herd size were also collected.

### Identification of risk factors of the disease

Univariate analysis and Chi square (χ^2^) test using a confidence interval of 95% at a *p*-value of ≤ 0.05 was used to identify the association between the potential individual risk factors and *Brucella* seropositivity. Risk factors associated with the disease were identified using multivariable logistic regression analysis of risk factors for *Brucella* seropositivity in cattle and small ruminants. Variables with a *p*-value ≤ 0.25 from the univariate analysis were included in the multivariable analysis. The backward stepwise (Wald) model was used and the validity of the test was assessed by computing Hosmer-Lemeshow goodness-of-fit.

### Data management and statistical analysis

The study used both qualitative and quantitative methods of data collection and analysis. Data was analyzed using Statistical Package for Social Sciences (SPSS) version 20. Descriptive statistics was run to obtain the frequency distribution and percentages and univariate analysis was computed to identify association between variables.

## Results

### Socio-demographic characteristics of studied pastoralist

A total of 143 pastoralists comprising females 9.0% (13/143) and males 91.0% (130/143) were included in the study. The majority of the participants were single 68.0% (97/143) and had not attended formal education 84% (120/145). The age category “18–25 years old” 63.0% (90/143) was the majority, followed by the age group ˃32 years old 22.3% (32/143).

### Overall seroprevalence of brucellosis in different animal species

A total of 143 human sera and 843 livestock comprising cattle (478), sheep (86) and goats (279), of different ages and sexes were screened for anti-*Brucella* antibodies. The seroprevalence in humans revealed 4.9% (7/143) and 4.2% (6/143) based on series testing using RBPT and c-ELISA, respectively. A seroprevalence of 21.7% (104/478) and 11.8% (33/278) based on RBPT were revealed in cattle and goats respectively. In contrast, c-ELISA revealed a seroprevalence of 21.3% (102/478) and 11.8% (33/278) in cattle and goats, respectively. All the 86 serum samples from sheep tested on RBPT and further subjected to c-ELISA were found to be negative as shown in Table 1.

**Table 1:** Overall seroprevalence of brucellosis in humans, cattle, goats and sheep based on serological tests.

Species	Number of sera tested	Seroprevalence	
		Total number of RBPT positive reactors n (%)	Total number of c-ELISA positive reactors n (%)
Human	143	7 (4.8)	6(4.2)
Cattle	478	104 (21.7)	102 (21.3)
Goats	279	33 (11.8)	33(11.8)
Sheep	86	0 (0.0)	0 (0.0)

### Seroprevalence of brucellosis in cattle camps

The seroprevalence in the cattle camps of Terekeka County was 100.0% (13/13) compared to Juba County which was 50.0% (2/4) as shown in Table 2. The within camps seroprevalence varied between 0.0% to 38.4%. The following species of livestock were sampled from the camps, cattle at 56.7%, followed by goats at 33.1% and sheep at 10.2%. Cattle were the dominant species in the camps of Terekeka County 89.5% (428/478) compared to Juba County 10.5% (50/478).

**Table 2:** Seroprevalence of brucellosis in different Counties, Payams and cattle camps, Central Equatoria State, South Sudan.

		number of animals tested (Positive)			
County	Cattle camp	Cattle	Goats	Sheep	Total
Terekeka	Bukoworpio	30(11)	52(50	18(0)	100 (16)
	Pawe	13(5)	0	0	13 (5)
	Logurak	31(4)	0	0	31 (4)
	Lopipiyo	65(20)	6(1)	1(0)	72 (21)
	Gwondolo	26(7)	0	0	26 (8)
	Libina	32(8)	0	0	32 (8)
	Nadula	20(7)	20(1)	0	40 (8)
	Pojulu	0	49(9)	1(0)	50 (9)
	Wun-mabior	30 (5)	30(6)	0	60 (11)
	Temi	91(6)	0	0	91 (6)
	Jongor	49(12)	0	0	49 (12)
	Jebel Namusia	0	43(2)	4(0)	47 (2)
	Sure	41(5)	19(0)	0	60 (5)
Juba	Gerekek	17(6)	44(9)	13(0)	74 (15)
	Dura	33(8)	1(0)	0	34 (7)
	Toch Manga	0	5(0)	21(0)	26 (0)
	Yasin farm	0	10(0)	28(0)	38 (0)
		**478(104)**	**279(33)**	**86(0)**	**843(137)**

### Risk factors associated with *Brucella* sero-positivity in humans, cattle and small ruminants in CES, South Sudan

#### Univariate logistic regression analysis

All the variables investigated in the univariate analysis of risk factors in cattle were significantly associated with *Brucella* seropositivity as shown in Table 3: sex (OR:4.5, 95% CI: 2.2–8.9, *p*<0.001), age (OR:6.6, 95% CI: 2.3–19.1, *p*<0.001), abortion history (OR:3.1, 95% CI: 1.8–5.2, *p*<0.001), retained placenta (OR:2.5, 95% CI: 1.4–4.4, *p* = 0.001), parity (OR:2.3, 95% CI: 1.1 - 4.7, *p* = 0.02) and reproductive status (category “dry” OR:3.329, 95%CI: 1.598–6.934, *p* = 0.001). The analysis shows that females have a significantly higher likelihood of testing positive compared to males, as indicated by the low *p-*value (<0.001). It was further established that individuals over 5 years old had the highest likelihood (OR: 6.6) of testing positive, followed by those aged 2–5 years old.

**Table 3:** Univariate analysis of potential risk factors associated with *Brucella* seropositivity in cattle in Central Equatoria State, South Sudan.

Variable	Category	No. sampled	No. positive	OR	95% CI	*p*-value
Sex	Male	132	10			
	Female	346	93	4.485	2.256–8.915	< 0.001
Age	≥ 1year old	75	4			
	2–5 years old	201	44	4.975	1.721–14.376	0.003
	>5 years old	202	55	6.641	2.315–19.050	< 0.001
Abortion	No	264	56			
	Yes	82	37	3.054	1.806–5.166	<0.001
Retained Placenta	No	281	65			
	Yes	65	28	2.515	1.431–4.419	0.001
Reproductive status	Not produced	91	17	-	-	-
	Pregnant	37	12	2.089	0.878–4.972	0.096
	Lactating	158	38	1.378	0.726–2.617	0.326
	Dry	60	26	3.329	1.598–6.934	0.001
	NA	132	10	-	-	-
Parity	Not produced	90	17	-	-	-
	Produced once	62	16	1.494	0.687–3.245	0.311
	Produced twice	74	26	2.326	1.142–4.739	0.020
	Produced more than twice	120	34	1.698	0.877–3.286	0.116

Nevertheless, in the univariate analysis of risk factors associated with *Brucella* seropositivity in small ruminants, animal species (χ^2^ = 11.183, *p*-value 0.001) and parity level (χ^2^ = 10.394, *p* = 0.034) were found to be associated with *Brucella* seropositivity as showed in Table 4. The risk of occurrence of brucellosis in goats is higher compared to sheep as supported by the low p-value (<0.001). In humans, blowing air through cow’s vagina (OR: 1.4, 95% CI: 0.782–2.434, *p* = 0.035) was the only variable found to be significantly associated with *Brucella* seropositivity at the univariate analysis of risk factors as shown in Table 5.

**Table 4:** Univariate analysis of potential risk factors associated with *Brucella* seropositivity in small ruminants.

Variable	Category	No. sampled	No. positive	χ^2^	*p*-value
Sex	Male	64	2	3.303	0.069
	Female	301	31		
Age	≤ 1year old	97	5	2.426	0.119
	˃ years old	268	28		
Animal species	Goats	279	33	11.183	0.001
	Sheep	86	0		
Retained placenta history	Yes	16	1	3.640	0.162
	No	285	30		
	N/A	64	2		
Abortion history	Yes	39	4	3.303	0.192
	No	262	27		
	N/A	64	2		
Reproductive status	Not produced	25	0	8.043	0.090
	Pregnant	64	7		
	Lactating	182	19		
	Dry	30	5		
	NA	64	2		
Parity level	Not produced	26	0	10.394	0.034
	Produced once	38	2		
	Produced twice	69	6		
	Produced more than twice	168	23		
	NA	64	2		

**Table 5:** Univariate analysis of potential risk factors associated with *Brucella* seropositivity in humans.

Variable	Category	No. Sampled	No. positive	OR	95%CI	*p*-value
Gender	Male	130	5	2.083	0.225–19.321	0.518
	Female	13	1	-	-	-
Age	18–25 years old	90	4	-	-	-
	25–32 years old	21	2	2.263	0.386–13.268	0.235
	˃ 32 years	32	0	0.000	0.000 -	0.998
Marital status	Single	97	3	2.425	0.469–12.528	0.290
	Married	40	3	-	-	-
Educational level	No formal education	120	6			0.753
	Primary education	16	0	0.000		
	Secondary education	6	0	0.000		
	Tertiary	1	0	0.000		
Occupation	Pastoralist	130(6)	6			0.960
	Farmer	8(0)	0			
	Butcher	1(0)	0			
	Student	2(0)	0			
	Other	2(0)	0			
Consumed raw meat	Yes	124(6)	6	0.86	0.805–0.921	0.327
	No	19(0)	0	-	-	-
Consumed raw milk	Yes	135(6)	6	0.942	0.903–0.982	0.542
	No	8(0)	0			
Blowing through vagina	Yes	130	4	1.4	0.782–2.434	0.035
	No	13	2	-	-	-
Herd size	50–100	5	0	-	-	-
	Above 100	138	131	0.583	0.362–0.941	< 0.001

### Multivariable logistic regression analysis

In cattle, all the six variables from the univariate analysis were included in the multivariable model. The multivariable logistic regression model identified sex, age, and abortion history as statistically significant factors of *Brucella* seropositivity in cattle as shown in Table 6. The odds of seropositivity were nearly threefold (OR: 2.8; 95% CI: 1.3–5.8, *p* = 0.006) higher in cows compared to bulls. Older cattle over two years had higher odds of *Brucella* seropositivity than young animals (OR: 3.5, 95% CI: 1.2–10.3-, *p* = 0.025). Cows with a history of abortion had higher odds of *Brucella* seropositivity (OR: 2.8, 95% CI: 1.6–4.7, *p*: <0.001).

**Table 6:** Multivariable logistic regression analysis of risk factors for *Brucella* seropositivity in cattle.

		Multivariate analysis of risk factors in cattle		
**Variable**	**Category**	**OR**	***p*-value**	**95% CI**
Sex	Male			
	Female	2.783	0.006	1.346–5.755
Age	< 1year old			
	2–5 years old	3.463	0.024	1.174–10.211
	> 5years old	3.474	0.025	1.168–10.328
Abortion history	No*			
	Yes	2.781	<0.001	1.631–4.739

The Hosmer-Lemeshow goodness-of-fit test showed that the model fairly fitted the data (χ^2^ = 10.281, *p*-value: 0.113).

However, in small ruminants, none of the variables was found to be statistically significant (*p* < 0.05) at the multivariate analysis with *Brucella* seropositivity.

## Discussion

This study has for the first time revealed seroprevalence of brucellosis in, goats, and the pastoral communities, and the absence of seropositive sheep in CES, South Sudan. The study revealed higher seroprevalence of brucellosis in cattle than in goats and identified the following risk factors; age, sex and previous history of abortion as significantly associated with *Brucella* seropositivity. The current seroprevalence of brucellosis among the pastoral community was 4.1% (6/143) based on c-ELISA performed on RBPT seropositive sera. This seroprevalence is lower compared to the finding of [11] in Wau, Western Bahr el Ghazal State (WBeGS), who revealed a seroprevalence of 33.3% of brucellosis among herders based on c-ELISA. Similarly, [13] reported a seroprevalence of 3.7% based on indirect ELISA in nomadic pastoralists in Chad. The variations on brucellosis seroprevalence could be attributed to spatial and temporal features, animal husbandry practices, pastoralists lifestyles, availability of veterinary services, control programs and test methods used [8].

In South Sudan, livestock production systems are categorized as pastoral and agro-pastoral. A variety of livestock species including cattle, sheep and goats are reared collectively and kept in camping called ‘cattle camps’. [21]. The dominant species kept is cattle, followed by goats and to a lesser extent the sheep as this correlate with number of samples collected in this study.

In cattle, the study revealed higher seroprevalence, 21.3% (102/478), compared to goats, 11.8% (33/279). Comparably, a high seroprevalence in cattle has been reported in pastoralists setting in Chad [13]. This finding is also in agreement with [28], who reported a significantly higher prevalence in cattle than in goats in Tanzania. In Kagera ecosystem in Tanzania, cattle were more at risk of contracting *Brucella* infection than goats [29]. Comparatively, the seroprevalence reported in cattle in this study is less compared to the 25.3% (86/340) reported by [20] and 29.3% (147/502) by [18] in South Sudan. However, another study reported a higher individual animal seroprevalence of 30.8% (88/285) and a lower herd prevalence of 77.7% in Kasulu district, Tanzania compared to the findings of this study [30].

The high seroprevalence in cattle could be due to their dominance in the cattle camps in the study area. In South Sudan, cattle are kept for prestige, and the pastoralists rarely contemplate selling or culling them out. Hence, cattle harbouring *Brucella* could have a chance of living longer than small ruminants in the cattle camps and would continue shedding infection given that the seroprevalence rises with age.

The study also revealed a seroprevalence of 11.8% (33/279) in goats. The seroprevalence was high in female goats 8.5% (31/365) compared to male goats 0.5% (2/365). Similarly, the prevalence of this study is in agreement with [31] who revealed higher prevalence of brucellosis in females 10.3% (31/301) compared to males 3.1% (2/64) in Arsi zone, Oromia, Ethiopia. Similarly, [32] reported a higher seroprevalence of brucellosis in female goats 1.4% (4/276) than males 0.0% (0/84) in Korahey zone, Somali regional state, eastern Ethiopia. The seroprevalence of goats in this study is also in agreement with a prevalence of 11.4% (35/307) reported by [16] on caprine in Khartoum State, Sudan. In contrast, [33] in Borona pastoral areas in southern Ethiopia reported higher prevalence 17.36% (137/789) of brucellosis in goats than the seroprevalence reported in this study.

Additionally, [9] reported a higher seroprevalence of 3.92% (13/332) in goats compared to 1.23% (1/81) in sheep in Karega District, Uganda. The fact that none of the well-established risk factors for *B*. *melitensis* infection in goats were found associated with seropositivity in goats in our study suggests that not *B*. *melitensis* but most likely *B*. *abortus* spilling over from cattle could be the cause for seropositivity in goats. Indeed, although reports of *B*. *abortus* infection in small ruminants are scarce, such infections have been reported worldwide [34].

This study revealed a 0.0% seroprevalence of brucellosis in sheep (0/86). This is in line with [31] in Ethiopia who reported a 0.0% seroprevalence of brucellosis. In West Africa, there is no report of *B*. *melitensis* infection in sheep and goats. Seropositivity in small ruminants was documented in Nigeria to be associated with *B*. *abortus* infection that had spilt over from infected cattle [35]. In Latin America, sheep are not significantly infected with *B*. *melitensis* even when kept in close contact with goats [36]. Moreover, they do not easily become infected with *B*. *abortus* [37]. This could be attributed to factors such as breed susceptibility, predominance species, husbandry practices and the self-limiting nature of the disease in sheep [38]. Reports from Egypt and Iran suggest that sheep are less susceptible to *B*. *abortus* infections than goats [34,39]. The fact that brucellosis seropositivity was not detected in sheep means that this species cannot be recognized as a source of human infection, which is an important epidemiological feature with implications in prospective One Health control measures. Moreover, it raises interesting questions regarding the aetiology of brucellosis in South Sudan. In this perspective, a point of concern is the potential emergence of *Brucella* species infecting non-preferential hosts.

In the analysis of the risk factors, the study identified a significant association of *Brucella* seropositivity with sex, age, and abortion history in cattle. A Higher prevalence of brucellosis was identified in female cattle, 19.5% (93/346) compared to males, 2.1% (10/132), and this difference was statistically significant (OR = 2.783, *p*-value < 0.006). This finding is in agreement with [14] who reported a significant association of *Brucella* seropositivity with sex on which female animals had higher level of exposure compared to males. Other researchers have reported similar findings of significant association of *Brucella* seropositivity in female animals [28,40]. This could be due to repeated exposure to *Brucella* spp. as female animals stay for longer periods in herds than males. Furthermore, the female reproductive tract provides a potential reservoir for the organism to propagate due to the presence of erythritol sugar which stimulates the growth of *Brucella* organism [38]. The current study also revealed that cows with a prior history of abortion had higher odds of *Brucella* seropositivity (OR: 2.8, 95% CI: 1.6–4.7, *p*<0.001). Our finding is in agreement with a previous study conducted in South Sudan [18] as well as with the findings from multiple studies [41–44] which reported an association of *Brucella* seropositivity with abortion. The study revealed that, older cattle over two years of age (OR: 3.5, 95% CI: 1.2–10.3-, *p* = 0.025) had higher odds of *Brucella* seropositivity than younger ones. This finding is in agreement with several studies [41,42] that also identified age as a risk factor for *Brucella* seropositivity in cattle. In contrast to the current study finding, another study revealed a higher odds of *Brucella* infection in the young compared to adults [45].

The fact that older cattle showed higher seropositivity to *Brucella* infection than the young ones could be attributed to continued exposure to pathogens, especially in the cattle camps where cattle are kept over long periods. The seroprevalence of brucellosis in the herds within cattle camps of Terekeka County was 100.0% compared to Juba County which was 50.0%. This finding is in agreement with [18] who reported herd seroprevalence based on c-ELISA at 61.4% and 90.0% for peri-urban Juba Town and rural Terekeka County cattle herds, respectively. Our results suggest that cattle are a reservoir of brucellosis in livestock, because of the highest seroprevalence found in this species, most likely due to *B*. *abortus*, its preferential host. The lower seroprevalence in goats suggests that *B abortus* may have spilled over from cattle to goats. The absence of seropositivity in sheep suggest that *B*. *melitensis* not endemic in this species and that *B*. *abortus* has not yet spilled over to the sheep due to the husbandry systems, with spatial and temporal segregation mainly between cattle and sheep.

The study had few limitations. The cross-sectional nature of this study limited its assessment on the causal relationship between identified risk factors and brucellosis seropositivity. The sample size of the small ruminants, especially the sheep was few compared to the number of cattle and goats. Hence, this has an effect on the generalization of the result. Additionally, vastness of the area, insecurity in some parts, remoteness of some cattle camps and the raining season have hindered accessibility. Despite these limitations, we feel that this study does provide an insight into the seroprevalence and risk factors of brucellosis in pastoralists and their livestock in Central Equatoria State, South Sudan.

## Conclusion

This study reports for the first time the seroprevalence of brucellosis in goats in South Sudan where it’s prevalence in livestock and pastoral community revealed its endemicity. Female cattle have a higher risk of infection compared to males. Previous history of abortion and older age cattle were significantly associated with *Brucella* seropositivity. Based on our findings, we recommend that control measures should be directed to cattle to reduce production losses and possible spillover to sheep and to prevent human contamination. Moreover, strategies for nationwide awareness campaigns and implementing the One Health approach are needed to mitigate brucellosis in South Sudan effectively. Efforts should be made to isolate *Brucella* spp. from cattle and goats to document that *B*. *abortus* has spilled over from its cattle reservoir to goats.

## Supporting information

S1 Dataset Cattle dataset used for analysis. (SAV)

S2 Dataset Shoat’s dataset used for analysis. (SAV)

S3 Dataset Human’s dataset used for analysis. (SAV)
